# A Case of Postpartum Ovarian Vein Thrombosis

**DOI:** 10.7759/cureus.2134

**Published:** 2018-02-01

**Authors:** Sidra Khalid, Aariez Khalid, Hamed Daw

**Affiliations:** 1 Internal Medicine Residency, Fairview Hospital, Cleveland Clinic, USA; 2 Bachelor of Science (biomedical Science), University of Guelph; 3 Department of Hematology and Oncology, Fairview Hospital, Cleveland Clinic, USA

**Keywords:** ovarian vein thrombosis, deep venous thrombosis, anticoagulation, antibiotics

## Abstract

Ovarian vein thrombosis (OVT) is an rare condition, which can present in the postpartum period. We present a case of a 35-year-old female who presented with right lower quadrant pain and fever. Her computed tomography (CT) abdomen revealed a dilated right ovarian vein with soft tissue attenuation material in its lumen that extended into the inferior vena cava, along with fat stranding of the surrounding soft tissues signifying thrombophlebitis. She was treated with enoxaparin and piperacillin-tazobactam, which lead to a resolution of the thrombus. Our case highlights the importance of prompt diagnosis and treatment of OVT in order to prevent morbidity and mortality.

## Introduction

Ovarian vein thrombosis (OVT) is a condition that occurs in 0.02-0.18% of pregnancies and is diagnosed on the right side in 80-90% of the affected patients. Patients with this condition present with symptoms of pelvic pain, fever, and a right-sided abdominal mass. Currently, the combination of anticoagulation and intravenous antibiotics is necessary for its treatment [1]. Therefore, we present a case of a postpartum female who was diagnosed with OVT and was effectively treated with enoxaparin and piperacillin-tazobactam.

## Case presentation

**Figure 1:**
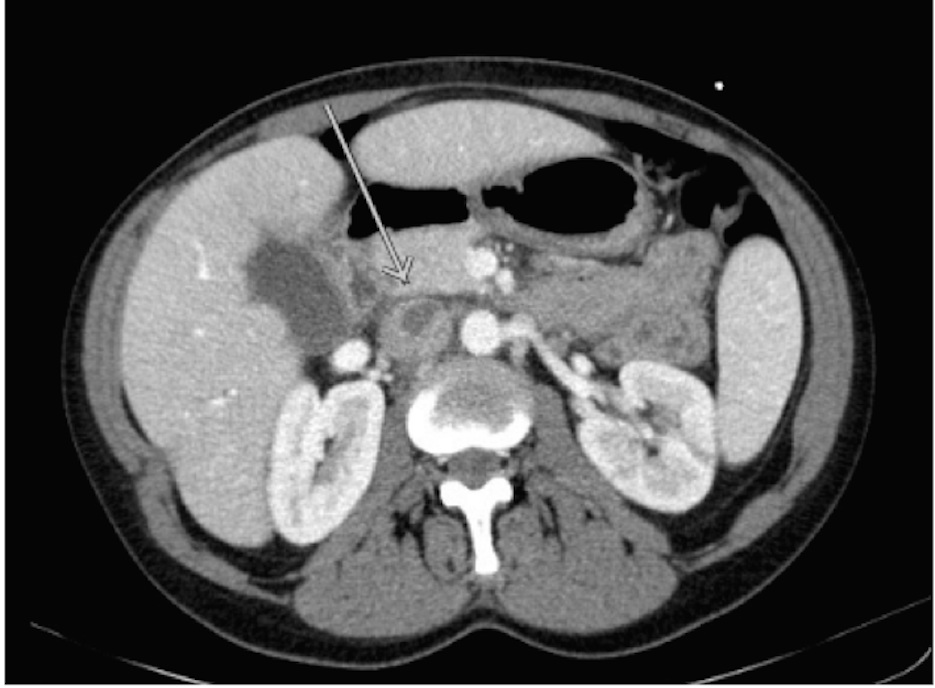
Extension of the right ovarian thrombus into the inferior vena cava at its insertion site (arrow).

A 35-year-old female gravida 9 para 5 presented to the emergency department with right lower quadrant pain and fever for two days. She was postpartum day 8 and had given birth to twins after an uncomplicated spontaneous vaginal delivery. She was an active smoker and smoked half pack per day for 20 years, had a history of substance abuse, and was a hepatitis C carrier. Her vital signs and physical examination was unremarkable except for tenderness on palpation of the right lower quadrant of the abdomen. Pelvic examination revealed uterus, cervical, and right adnexal tenderness. A transvaginal ultrasound was performed that showed a small amount of fluid and blood products within the endometrial cavity. Computed tomography (CT) of the abdomen and pelvis with contrast revealed a markedly dilated right ovarian vein containing abnormal soft tissue attenuation material throughout, with fat stranding along the course of the vein signifying thrombophlebitis. A right ovarian vein thrombus extending into the inferior vena cava at its insertion, measuring 1.5 cm was found (Figure 1). There was no past medical history or family history of hypercoagulability or blood clots, except for blood clots in a sister who was on oral contraceptive pills and an uncle who had cancer. With an initial diagnosis of septic thrombophlebitis, the patient was started on enoxaparin 70 mg every 12 hours and piperacillin-tazobactam. On day 4, piperacillin-tazobactam was stopped, as she was afebrile for 48 hours.

The option of bridging to warfarin was offered but due to the necessity of transportation for international normalized ratio (INR) monitoring it was declined by the patient. Rivaroxaban was not an option as she was breastfeeding. Therefore, it was recommended to continue enoxaparin for at least three months, with outpatient follow-up with hematology. The patient did not follow up as outpatient. She took enoxaparin for three months and then stopped by herself. After six months from her diagnosis of right ovarian vein thrombosis, she presented to the emergency department for right lower quadrant pain. Her transvaginal ultrasound was unremarkable. A CT of the abdomen and pelvis showed a normal caliber of the right ovarian vein with resolution of the intraluminal thrombus.

## Discussion

OVT occurs in about 1/600 to 1/2000 pregnancies. It presents as a triad of pain, fever, and abdominal mass [2]. In a recent study by Assal et al., out of 223 cases, there was an equal incidence of right and left OVT; however, in a peripartum state, right OVT was more common [3]. It is hypothesized that OVT commonly occurs on the right side because the right ovarian vein is longer than the left and it lacks competent valves [2]. The right ovarian vein enters the inferior vena cava at an acute angle, which makes it more susceptible to compression [4]. Furthermore, the dextrorotation of the enlarged uterus that occurs in pregnancy can cause compression of the right ovarian vein and the right ureter as they cross the pelvic rim, which causes stasis of blood leading to thrombosis [5]. During the postpartum period, there is anterograde flow in the right ovarian vein as compared to the retrograde flow in the left ovarian vein, which predisposes to right-sided thrombosis [4].

Diagnosing OVT is important to prevent morbidity and mortality. The gold standard for diagnosis is laparotomy, which was used in the past [5]. The most common imaging technique that is used is magnetic resonance angiography, which has the highest sensitivity and specificity that approaches close to 100%. Also, CT scan with intravenous contrast and color Doppler can also be used. CT scan with intravenous contrast has also shown a 100% sensitivity and sensitivity in some studies, therefore it should be used as an initial technique due to its cost effectiveness [4]. CT scan findings show a thick-walled enlarged ovarian vein with rim enhancement and central hypodensity [5]. If this disease is not diagnosed, then the ovarian vein thrombus could extend into the inferior vena cava or iliofemoral vessels, which can lead to pulmonary arterial embolization. Pulmonary embolism occurs in 25% of untreated OVT with a mortality as high as 4% [2]. Another complication that could be encountered with OVT is sepsis due to thrombophlebitis. Other complications of OVT include ovarian abscess, ovarian infarction, uterine necrosis, or ureteral compression [6].

The main treatment of OVT includes antibiotics and/or anticoagulation based on the clinical presentation. There are no specific guidelines in place for the duration of treatment. In a study by Wysokinsja et al., the duration of treatment with warfarin of lower deep venous thrombosis (DVT) was 5.3 months and of OVT was 6.9 months. Hence, guidelines for treating lower extremities DVT could be applied for treating OVT. Furthermore, if the patients have a hypercoagulable disorder then anticoagulation should be lifelong. A seven-day course of antibiotics can also be given when there is suspicion of septic thrombophlebitis [2].

## Conclusions

OVT is a rare clinical entity. A high index of suspicion is required for the prompt diagnosis of OVT, especially in any postpartum women presenting with lower abdominal pain. Through early diagnosis and appropriate treatment with anticoagulation and antibiotics, life-threatening complications could be prevented.
